# Characterizing neurocognitive impairments in Parkinson’s disease with mobile EEG when walking and stepping over obstacles

**DOI:** 10.1093/braincomms/fcad326

**Published:** 2023-11-28

**Authors:** Magda Mustile, Dimitrios Kourtis, Martin G Edwards, Simon Ladouce, Daniele Volpe, Manuela Pilleri, Elisa Pelosin, Gemma Learmonth, David I Donaldson, Magdalena Ietswaart

**Affiliations:** Psychology, Faculty of Natural Sciences, University of Stirling, Stirling, FK9 4LA, UK; The Psychological Sciences Research Institute, Université catholique de Louvain, 1348 Louvain-la-Neuve, Belgium; Psychology, Faculty of Natural Sciences, University of Stirling, Stirling, FK9 4LA, UK; The Psychological Sciences Research Institute, Université catholique de Louvain, 1348 Louvain-la-Neuve, Belgium; Department of Brain and Cognition, Leuven Brain Institute, KU Leuven, 3000 Leuven, Belgium; Fresco Parkinson Center, Villa Margherita, S. Stefano Riabilitazione, 36100 Vicenza, Italy; Fresco Parkinson Center, Villa Margherita, S. Stefano Riabilitazione, 36100 Vicenza, Italy; Ospedale Policlinico San Martino, IRCCS, 16132 Genova, Italy; Psychology, Faculty of Natural Sciences, University of Stirling, Stirling, FK9 4LA, UK; School of Psychology & Neuroscience, University of Glasgow, Glasgow, G12 8QQ, UK; School of Psychology and Neuroscience, University of St Andrews, St. Andrews, KY16 9AJ, UK; Psychology, Faculty of Natural Sciences, University of Stirling, Stirling, FK9 4LA, UK

**Keywords:** Parkinson’s disease, obstacle avoidance, gait, cognitive control, brain oscillations

## Abstract

The neural correlates that help us understand the challenges that Parkinson’s patients face when negotiating their environment remain under-researched. This deficit in knowledge reflects the methodological constraints of traditional neuroimaging techniques, which include the need to remain still. As a result, much of our understanding of motor disorders is still based on animal models. Daily life challenges such as tripping and falling over obstacles represent one of the main causes of hospitalization for individuals with Parkinson’s disease. Here, we report the neural correlates of naturalistic ambulatory obstacle avoidance in Parkinson’s disease patients using mobile EEG. We examined 14 medicated patients with Parkinson’s disease and 17 neurotypical control participants. Brain activity was recorded while participants walked freely, and while they walked and adjusted their gait to step over expected obstacles (preset adjustment) or unexpected obstacles (online adjustment) displayed on the floor. EEG analysis revealed attenuated cortical activity in Parkinson’s patients compared to neurotypical participants in theta (4–7 Hz) and beta (13–35 Hz) frequency bands. The theta power increase when planning an online adjustment to step over unexpected obstacles was reduced in Parkinson’s patients compared to neurotypical participants, indicating impaired proactive cognitive control of walking that updates the online action plan when unexpected changes occur in the environment. Impaired action planning processes were further evident in Parkinson’s disease patients’ diminished beta power suppression when preparing motor adaptation to step over obstacles, regardless of the expectation manipulation, compared to when walking freely. In addition, deficits in reactive control mechanisms in Parkinson’s disease compared to neurotypical participants were evident from an attenuated beta rebound signal after crossing an obstacle. Reduced modulation in the theta frequency band in the resetting phase across conditions also suggests a deficit in the evaluation of action outcomes in Parkinson’s disease. Taken together, the neural markers of cognitive control of walking observed in Parkinson’s disease reveal a pervasive deficit of motor–cognitive control, involving impairments in the proactive and reactive strategies used to avoid obstacles while walking. As such, this study identified neural markers of the motor deficits in Parkinson’s disease and revealed patients’ difficulties in adapting movements both before and after avoiding obstacles in their path.

## Teaser

Mustile *et al*. report that Parkinson’s disease patients showed impaired modulation of neural markers of proactive and reactive cognitive control while walking and stepping over obstacles. Using mobile EEG, the study revealed that Parkinson’s patients have difficulty both in the approach and once past the obstacle.

## Graphical Abstract

**Graphical Abstract:**
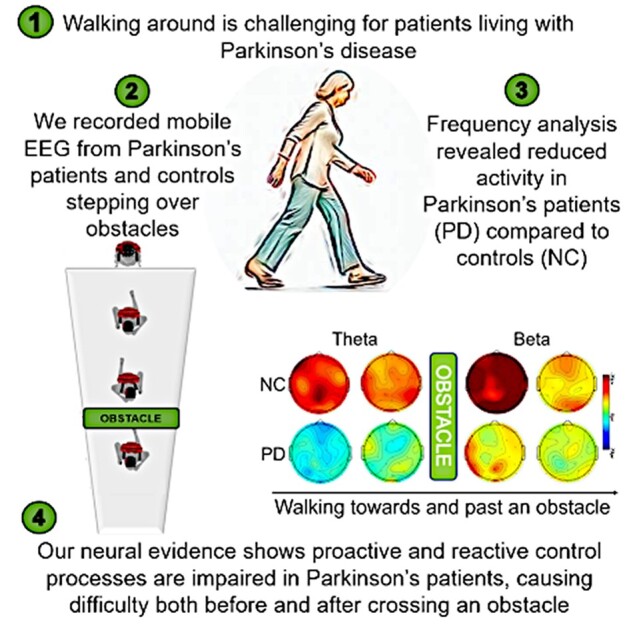

## Introduction

In the last decades, a growing body of evidence has demonstrated that both normal and challenging gait tasks involve cognitive control.^1,2^ Indeed, apparently simple daily living activities, such as walking while speaking on the phone or negotiating unexpected obstacles on the floor, require increased cognitive resources such as attention, planning of movements and monitoring online postural adjustments.^1,3^ Remarkably, our knowledge of the neural control of human gait is still limited, with inferences made largely on the basis of animal model research.^4-7^ This is mainly due to the limitations of brain imaging techniques, which present substantial constraints on investigations of the neurophysiological correlates of human gait. Instead, human gait has been mainly studied in laboratory settings while participants either imagine walking or perform minimal movements with their feet while lying in a scanner.^8-10^ Only recently, with the development of new portable devices, such as the mobile EEG and functional near-infrared spectroscopy, has it been possible to directly investigate the neural circuits supporting gait control in humans. A major finding of these initial investigations shows the coupling between gait and rhythmical oscillatory activity in the brain,^11-14^ which elucidated cortical contributions to the control of gait adjustments.^15-17^ The present study extends this approach to examine the neural correlates of changes in gait control in Parkinson’s disease.

The loss of the ability to control gait adjustments represents one of the main risk factors for falling and hospitalization in the elderly and in patients with neurological disorders, such as Parkinson’s disease. Gait impairments are particularly frequent in Parkinson’s disease patients.^18,19^ It is estimated that they affect ∼78% of Parkinson’s disease patients, and they can be continuously present from the early stage of the disease or episodic and then worsen with its progression.^20^ Typically, Parkinson’s disease patients with gait disturbances exhibit reduced gait speed, shorter stride length, stooped posture, reduced arm swing, increased gait asymmetry, poor postural control and impaired rhythmicity.^21-24^ Moreover, gait variability (e.g. fluctuations in gait cycle consistency) and the loss of dynamic structure of gait patterns provide predictive markers of cognitive deficits in Parkinson’s disease.^25,26^ Cortical markers of cognitive and motor control in Parkinson’s disease have been investigated in isolation, using tasks with relatively low ecological validity. To better understand cognitive and neural processes underlying dynamic movements, such as when walking and dealing with obstacles, research is required that targets natural walking behaviours. The negative impact that gait impairments have on the quality of life of Parkinson’s disease patients concerns not only the independence and the autonomy of the patient in daily living activities but also the risk of falls, which increases the rate of hospitalization and mortality in this population.^27,28^ Therefore, it is necessary to develop new neuroscientific methods to investigate neural markers of cognitive control during the performance of natural dynamic movements, in circumstances that more closely resemble real-life scenarios.^20^

Notably, in a previous study employing mobile EEG, we successfully identified neural markers of dynamic obstacle avoidance in neurotypical participants.^17^ We provided ‘real-world’ evidence that oscillations in the theta (4–7 Hz) frequency range reflect proactive control strategies that are engaged during naturalistic walking. More specifically, we identified a significant power increase in theta oscillations when participants had to adjust their gait to avoid unexpected obstacles. This change in EEG spectral power was not visible when participants could plan the gait adjustment (i.e. when obstacles were already visible from the outset), or when they did not need to adjust their gait (i.e. when not encountering any obstacles on their path). Our previous work corroborates the premise that theta oscillations signal proactive control processes and is furthermore consistent with evidence showing theta power increases when participants performed demanding cognitive tasks.^29-31^ For example, Cooper *et al*.^29,30^ reported an increase in theta power during the preparation of a switched response, suggesting that theta power increases signal the preparation for a change in the task and the updating of the goal of the response. Similarly, Liegel *et al*.^31^ observed increases in theta oscillations when participants prepared for a prioritization task, i.e. they had to concentrate only on one task at a time, as signalled by a cue, and that behavioural performance was better when the task was signalled as important. Additionally, and in line with theoretical accounts of proactive motor control,^32^ we previously showed^17^, on the basis of the temporal properties of EEG data, that cognitive processing of motor planning prior to encountering an obstacle can be divided into two distinct components: an early component, reflected in theta (4–7 Hz) power increase, and a late component, indexed by a decrease in beta (13–35 Hz) power, reflecting the preparation and the effective implementation of the behavioural adjustment. Moreover, we were able to demonstrate neural signals associated with reactive control processes that are required after an obstacle is overcome, reflected in beta power increases. As previously reported, for beta power, an increase is observed during movement recovery immediately after a challenging aspect of a motor task, specifically after a balance perturbation, possibly indicating adaptation processes and the integration of sensorimotor information required to maintain body stability during a task.^33^ This is, furthermore, in line with the premise that such a beta power increase, also known as beta rebound, indexes the recalibration of the motor system after a change in the motor response.^34^

Taken together, existing evidence strongly suggests a relationship between motor adjustments while walking and cognitive control mechanisms, which are both thought to be impaired in Parkinson’s disease participants. Indeed, several studies using traditional laboratory paradigms have highlighted that Parkinson’s disease participants exhibit less theta power modulation compared to neurotypical controls (NC) in tasks requiring adaptation to interference.^35,36^ Specifically, Parkinson’s disease participants show less theta power modulation during the habituation to novel stimuli^29^ and when responding to conflict tasks.^36^ For example, Singh *et al*.^36^ showed that Parkinson’s disease patients exhibited attenuated mid-frontal theta power when responding to incongruent trials and when processing errors compared to healthy participants in a Simon task.^37^ Related findings have been reported in a study investigating cortical activity during pedalling, with a stationary mini pedal exerciser, in Parkinson’s disease patients with and without freezing of gait.^38^ EEG data suggested that Parkinson’s disease patients with freezing of gait exhibit attenuated mid-frontal theta power compared to Parkinson’s disease participants without freezing of gait. These findings are, furthermore, in line with neuroimaging studies showing that the connectivity within the frontal–basal ganglia network is altered in Parkinson’s disease patients with freezing of gait, suggesting that gait disturbances might be associated with cognitive control impairments,^39,40^ which are thought to be reflected in the attenuated theta rhythm.^41^ In addition, Parkinson’s disease participants also exhibit deficits in action planning mechanisms, as shown by paradigms involving upper limb movements, which are reflected in reduced modulation of oscillations in the beta frequency band.^42,43^ Indeed, diminished beta power in Parkinson’s disease compared to neurotypical controls has been observed during motor preparation and response inhibition of fingers,^42^ as well as during the execution of rhythmical finger movements,^43^ reflecting altered patterns of cortical activity that have been found to correlate with gait impairments.^44,45^

Overall, current findings suggest that both proactive and reactive cognitive control of walking (reflected in theta and beta oscillations) might be compromised in Parkinson’s disease, especially when patients are required to adjust their gait suddenly, in response to unexpected changes, such as the sudden appearance of an obstacle on the floor. Avoiding obstacles requires motor programmes to be flexibly adapted while maintaining postural stability and balance, which are compromised in Parkinson’s disease.^46^ To date, however, the neural correlates of cognitive control of walking have not been investigated in participants with Parkinson’s disease under naturalistic walking conditions. Consequently, the main aim of the present study was to demonstrate how proactive and/or reactive processes of dynamic real-world movements are impaired in non-demented Parkinson’s disease during walking. These neurophysiological signals are expected to emerge when participants with Parkinson’s disease are required to adjust their gait and adapt their movements to avoid obstacles on the floor. For this purpose, we used a design based on our previous study,^17^ in which participants walked along a path and had to step over expected and unexpected obstacles projected on the floor. As in our previous study, we used mobile EEG, which we have shown to be a reliable tool to capture neural markers of proactive and reactive control while participants move through the environment. Building on our previous findings, we uniquely test whether participants with Parkinson’s disease exhibit a deficit when planning an online adjustment to face an unexpected obstacle displayed on the floor, which should be reflected by attenuated theta activity compared to neurotypical participants. Moreover, we also test whether participants with Parkinson’s disease show compromised motor preparation and reactive control processes after motor adjustments, reflected in attenuated cortical beta modulation compared to neurotypical participants. By employing mobile EEG, we are able to characterize changes in cognitive control that occur in Parkinson’s disease while participants walk naturally in a real world scenarios, stepping over expected and unexpected obstacles on the floor.

## Materials and methods

### Participants

Sixteen participants with idiopathic Parkinson’s disease without cognitive decline were recruited from the Fresco Parkinson Center of Villa Margherita, Santo Stefano Rehabilitation (Vicenza, Italy). Parkinson’s disease diagnoses were confirmed by clinicians and neurologists (D.V., M.P.) according to the Parkinson’s Disease Society Brain Bank clinical diagnostic criteria. A group of 18 neurotypical control participants took part in the study on a voluntary basis. For both groups, we recruited all eligible participants (see Supplementary Section S1.1 for inclusion criteria) who agreed to take part between May and August 2021. As part of recruitment, patients were screened on their ability to meet the demands of the task, including the length of time they would be walking when taking part (22 eligible patients were deemed not suited to participation and were not recruited to the study). Of those recruited, two participants from the Parkinson’s disease group and one participant from the control group were excluded due to excessive EEG artefacts (see the ‘EEG acquisition and pre-processing’ section of the Supplementary material, for detailed exclusion criteria of EEG artefacts; see Table 1 for demographics). The Hoehn and Yahr^47^ and Unified Parkinson's Disease Rating Scale^48^ (UPDRS) scores are those recorded in the medical notes on admission. All participants completed the Mini-Mental State Examination, and none of them scored under the cut-off criteria of ≥24 (as per Measso *et al*.^49^). Participants with Parkinson’s disease also performed a series of tasks to assess disease severity at the time of testing (see Table 1 for all clinical details). Participants with Parkinson’s disease were tested in the ON phase of the medication state, between 0.5 and 2 h after the last consumption of medication (see Table 1 for details). The study was approved by the local research ethics committee. All participants gave their written informed consent after being provided with a detailed explanation of the study protocol and aims.

**Table 1:** Summary of participant details

	Parkinson’s disease (*n* = 14), mean ± SD	Controls (*n* = 17), mean ± SD	*P* ^g^
Gender (total female/male/other)	5/9/0	11/6/0	
Age range (years)	50–79	50–71	
Mean age (years ± SD)	71.6 (±6.1)	68.8 (±6.1)	
Mean disease duration (years ± SD)	8.9 (±7.3)		
Hoehn and Yahr^a^ range (Stages 1–5)	2–3		
Mean Hoehn and Yahr^a^ (stage ± SD)	2.64 (±0.49)		
UPDRS^b^ part II (mean ± SD)	13.43 (±8.2)		
UPDRS^b^ part III (mean ± SD)	25.5 (±14.9)		
Levodopa dose^c^	529.14 (±309.29)		
Freezing of gait^d^ (*n*)	3		
MMSE^e^ (mean ± SD)	27.57 ± 1.98	29.41 ± 0.71	0.148
Education in years (mean ± SD)	9 ± 4.24	14.88 ± 5.38	0.060
Speed no adjustment condition (m/s)	0.81 ± 0.17	1.088 ± 0.07	<0.001
Speed preset adjustment condition (m/s)	0.82 ± 0.12	1.056 ± 0.14	<0.001
Speed online adjustment condition (m/s)	0.81 ± 0.15	1.158 ± 0.37	0.003
Stanford Sleepiness Scale^f^—Block 1	2.43 ± 1.78	2.35 ± 1.57	0.901
Stanford Sleepiness Scale^f^—Block 2	2.71 ± 1.77	2.35 ± 1.41	0.532
Stanford Sleepiness Scale^f^—Block 3	3.50 ± 2.06	2.53 ± 1.46	0.137
Stanford Sleepiness Scale^f^—Block 4	4 ± 2.03	2.76 ± 1.52	0.063

^a^Hoehn and Yahr Scale.^46^

^b^United Parkinson’s Disease Rating Scale (UPDRS).^47^

^c^Freezing of gait diagnosed by clinicians.

^d^Levodopa equivalent daily dose^50^ at the time of testing.

^e^Mini-Mental State Examination.^51^

^f^Stanford Sleepiness Scale^52^ assessed at the end of each block.

^g^Independent sample *t*-tests (*P*).

### Material and procedure

**Figure 1:**
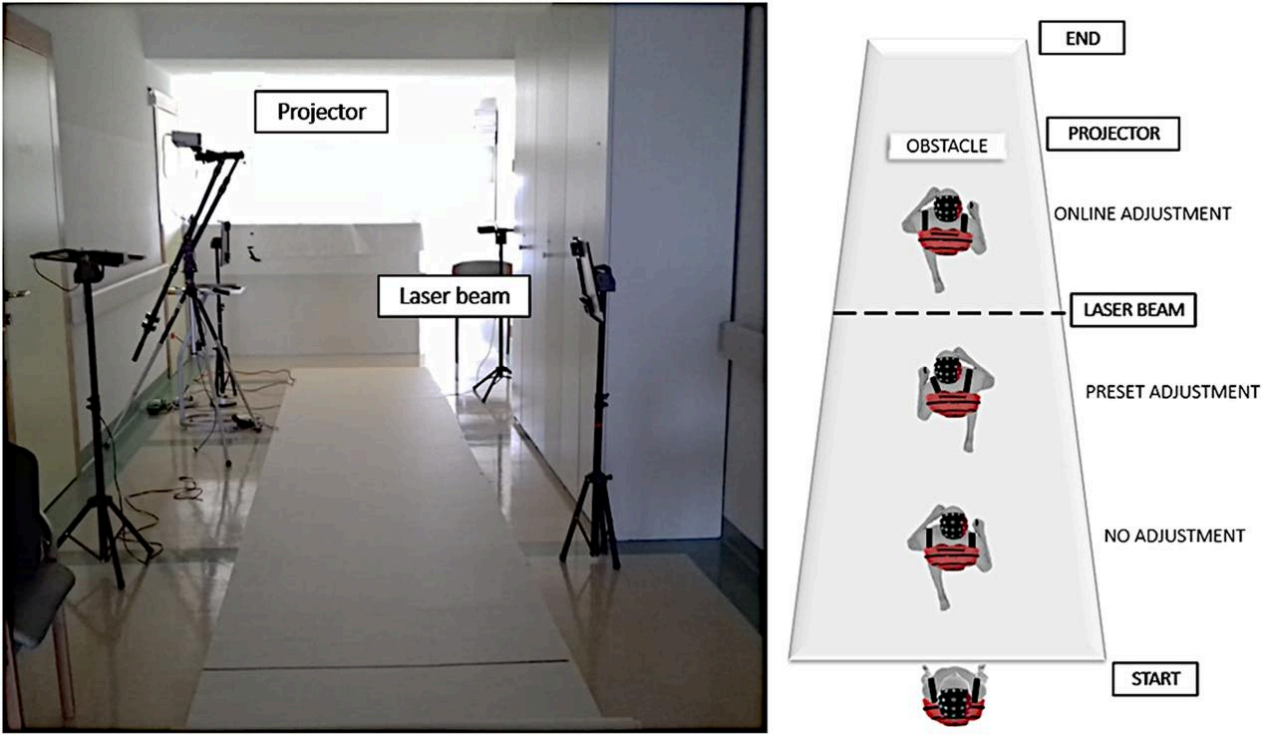
**Illustration of the task.** Left: picture of the setup. Right: illustration of the task across different conditions. In the no adjustment condition, after the start, participants walked through the path without obstacles. In the preset adjustment condition, the obstacle was visible from the beginning of the walking path in each trial. In the online adjustment condition, the obstacles appeared randomly only after the participants passed through the laser beam. During each trial, the onset of walking (start), the passage through the motion sensors (‘approach’), the obstacle avoidance (‘crossing’) and the cessation of walking (‘stop’) were recorded, providing the temporal markers for the EEG data for the planning phase (before the obstacle was encountered, from the ‘approach’ to ‘crossing’) and a resetting phase (after the obstacle was encountered, from ‘crossing’ to the ‘stop’). Additional information regarding the setup can be found in Supplementary Section S1.1.

We employed an experimental design similar to our previous study.^17^ The experiment included three different walking conditions. Participants walked along a 6 m-long carpet, passing through a series of motion sensors, which controlled the presentation of obstacles (projected onto the floor as white rectangles of 40 × 80 cm that had to be stepped over). The participants were instructed to wait for a start signal given by the experimenter, before walking straight along the path at a comfortable pace. In the ‘no adjustment’ condition, no obstacle was presented, and participants freely walked along the path. In the ‘preset adjustment’ condition, the participants were asked to walk and step over the obstacle, which was present throughout the trial, projected at a fixed location 250 cm from the first laser beam. In the ‘online adjustment’ condition, walking through the laser beam triggered the presentation of an obstacle projected at a distance of either 160 or 310 cm from the laser beam. The participants were asked to walk the path and step over any obstacle that could be presented in front of them. In all conditions, participants were instructed to stop walking when they reached the end of the path, turn around and wait for the go signal to start another trial. The video projector and the motion sensors were placed at the side of the path to allow the obstacle projection in both directions of walking (see Fig. 1). Participants completed a total of 160 randomized trials divided into four experimental blocks. Each block lasted around 5 min. Participants were given 5–10 min breaks between experimental blocks and encouraged to request additional breaks during each block if needed. Any systematic influence of fatigue on the data was minimized through randomization of condition order across participants. Levels of alertness were assessed using the Stanford Sleepiness Scale at the end of each block (see Table 1). The overall experimental session lasted ∼90 min, including EEG preparation, recording and breaks between experimental blocks.

### EEG acquisition and pre-processing

EEG data were recorded from 32 Ag/AgCl electrodes connected to a portable amplifier (ANT-neuro, Hengelo, The Netherlands). Electrodes were positioned according to the International 10–20 system (see Supplementary Section S1.2, for details regarding montage, recording, processing pipelines, independent component analysis and artefact rejection). Components exceeding a 90% probability of being eye, muscle, heart, line noise and channel noise were rejected. Only brain independent components with dipoles located inside the head and a residual variance lower than 15% were kept.

After rejection of artefactual components, the remaining data were segmented into epochs ranging from −5000 to 3000 ms around obstacle avoidance markers (i.e. the obstacle ‘crossing’ event, which was defined as time 0). In the no adjustment condition, the ‘crossing’ event was manually added at a fixed latency of 2000 ms from the ‘approach’ event (as in Mustile *et al*.^17^). To address the inter-trial variance in walking speed across groups and conditions, single-trial spectrograms were time warped to the median latency (across participants) of the ‘approach’ event using linear interpolation. Epochs were further visually inspected to identify trials that still appeared to be contaminated by prominent muscle artefacts, and these were manually removed. For the control group, an average (mean ± SD) of 29.82 ± 6.39 epochs was included in the analysis, whereas for the Parkinson’s disease group, an average (mean ± SD) of 28.93 ± 8.53 epochs was included in the analysis. Event-related spectral perturbations were obtained by computing the mean difference between single-trial log spectrograms for each channel, for each participant and the mean baseline spectrum, from −4500 ms preceding to 2000 ms following the obstacle stepping. The baseline corresponded to the mean activity of the overall epoch, and the interval was chosen to include the longest latency of the median of the ‘start’ (the onset of walking) across participants and conditions.

### Statistical analyses

The walking speed (m/s) was computed from the timestamp of the start and end events of each trial and averaged for both neurotypical and Parkinson’s disease participants for each condition (see Table 1). A mixed ANOVA and *post hoc* independent sample *t*-tests with Condition (no adjustment versus preset adjustment versus online adjustment) as a within-subject factor and Group (Parkinson’s disease versus neurotypical controls) as a between-subject factor were used to assess differences between groups, across conditions, using SPSS (IBM SPSS Statistics for Windows, Version 21.0). In addition, a repeated measures ANOVA with Condition (no adjustment versus preset adjustment versus online adjustment) as a within-subject factor (followed up with paired sample *t*-tests) was used to assess differences within groups. The significance threshold was set at alpha < 0.05.

### EEG analysis

In accordance with our previous investigation,^17^ we defined three regions of interest (ROIs): frontal (channels FC1, FC2 and Fz), central (channels CP1, CP2 and Cz) and posterior (channels P3, P4 and POz). Similar to our previous work,^17^ the planning and the resetting periods were divided into four successive ‘planning’ and two ‘resetting’ time windows, respectively. Each time-warped epoch of the planning phase was divided respectively into four consecutive time windows, whereas for the resetting, time windows were of 600 ms each. Repeated measures ANOVAs with within-subject factors of experimental condition (no adjustment versus preset adjustment versus online adjustment), time window (Time 1, Time 2, Time 3 and Time 4 for planning; Time 1 and Time 2 for resetting) and ROI (frontal versus central versus posterior) and a between-subject factor of group (Parkinson’s disease versus neurotypical controls) were performed to examine the power modulation across the planning and the resetting phases, separately for each frequency band. The significance level for the statistical analysis was set at *P* < 0.05. Where the sphericity assumption was violated, the Greenhouse–Geisser method was used to correct the degrees of freedom. *Post hoc* independent and paired sample *t*-tests were adjusted for multiple comparisons using Bonferroni correction.

## Results

### Behavioural effects: walking speed

**Figure 2:**
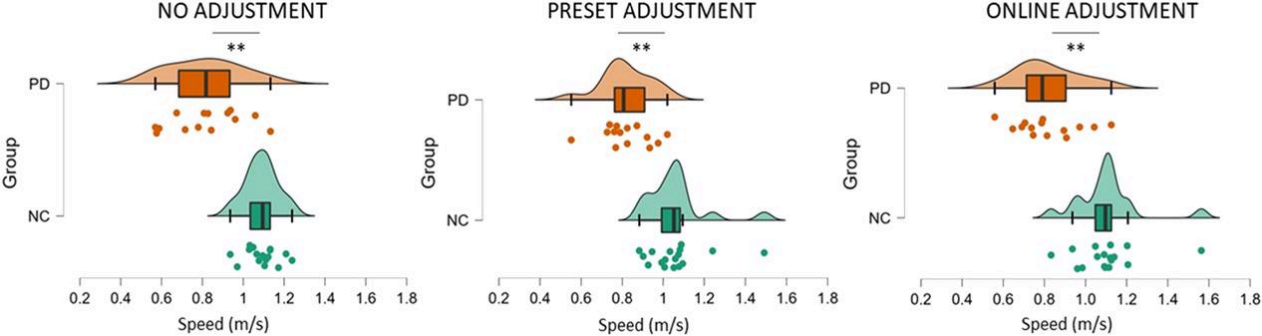
**Comparison of the speed (m/s) between neurotypical control (NC) and Parkinson’s disease (PD) groups across conditions.** The analysis revealed that participants with Parkinson’s disease were slower compared to neurotypical participants in the no adjustment condition (*t* = 5.376, *P* < 0.001), in the preset adjustment (*t* = 4.967, *P* < 0.001) and in the online adjustment conditions (*t* = 3.193, *P* = 0.003). Significant comparisons are flagged with the asterisks.

Analysis (ANOVA) of behavioural differences with regard to walking speed showed a main effect of group [*F*(1,30) = 31.148, *P* < 0.001, *η*_p_^2^ = 0.588] indicating that participants with Parkinson’s disease generally walked slower compared to control participants. A two-way interaction between group and condition [*F*(1,30) = 8.563, *P* < 0.001, *η*_p_^2^ = 0.235, Fig. 2] revealed that participants’ speed from the two groups differed in the three experimental conditions. *Post hoc* independent sample *t*-tests revealed that participants with Parkinson’s disease were slower than neurotypical control participants in the no adjustment condition [*t*(29) = 5.376, *P* < 0.001], in the preset adjustment [*t*(30) = 4.967, *P* < 0.001] and in the online adjustment conditions [*t*(30) = 3.193, *P* = 0.003]. As shown in Fig. 2, the difference between groups’ speed was higher in the online adjustment condition (mean ± SD = 0.34 ± 0.49), followed by the no adjustment condition (mean ± SD = 0.27 ± 0.34) and the preset adjustment condition (mean ± SD = 0.23 ± 0.33).

*Post hoc* paired sample *t*-tests of within-group effects did not reveal any significant differences in speed across conditions within either the Parkinson’s disease or the neurotypical control group (*P* > 0.05).

### EEG data

**Figure 3:**
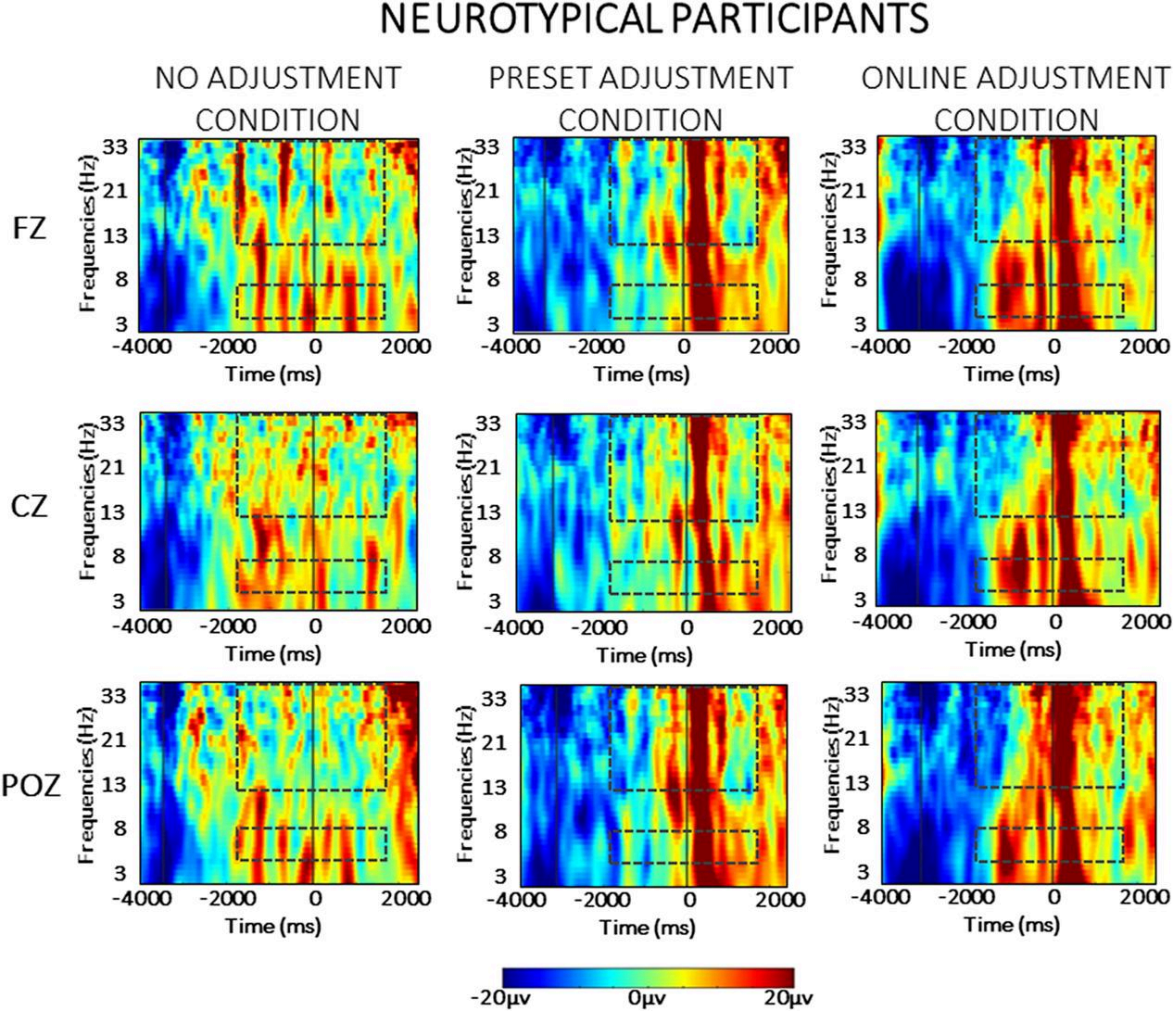
**Averaged time-warped spectrograms of representative midline channels (Fz, Cz and POz) in the neurotypical control group across conditions.** The vertical lines on the left (around −3000 ms) represents the start of the trial; the vertical line (at 0 ms) represents the obstacle crossing event. The two dashed squares in each figure highlight the time and the frequency bands of interest (i.e. theta at 4–7 Hz and beta at 13–35 Hz) in the analysis.

**Figure 4:**
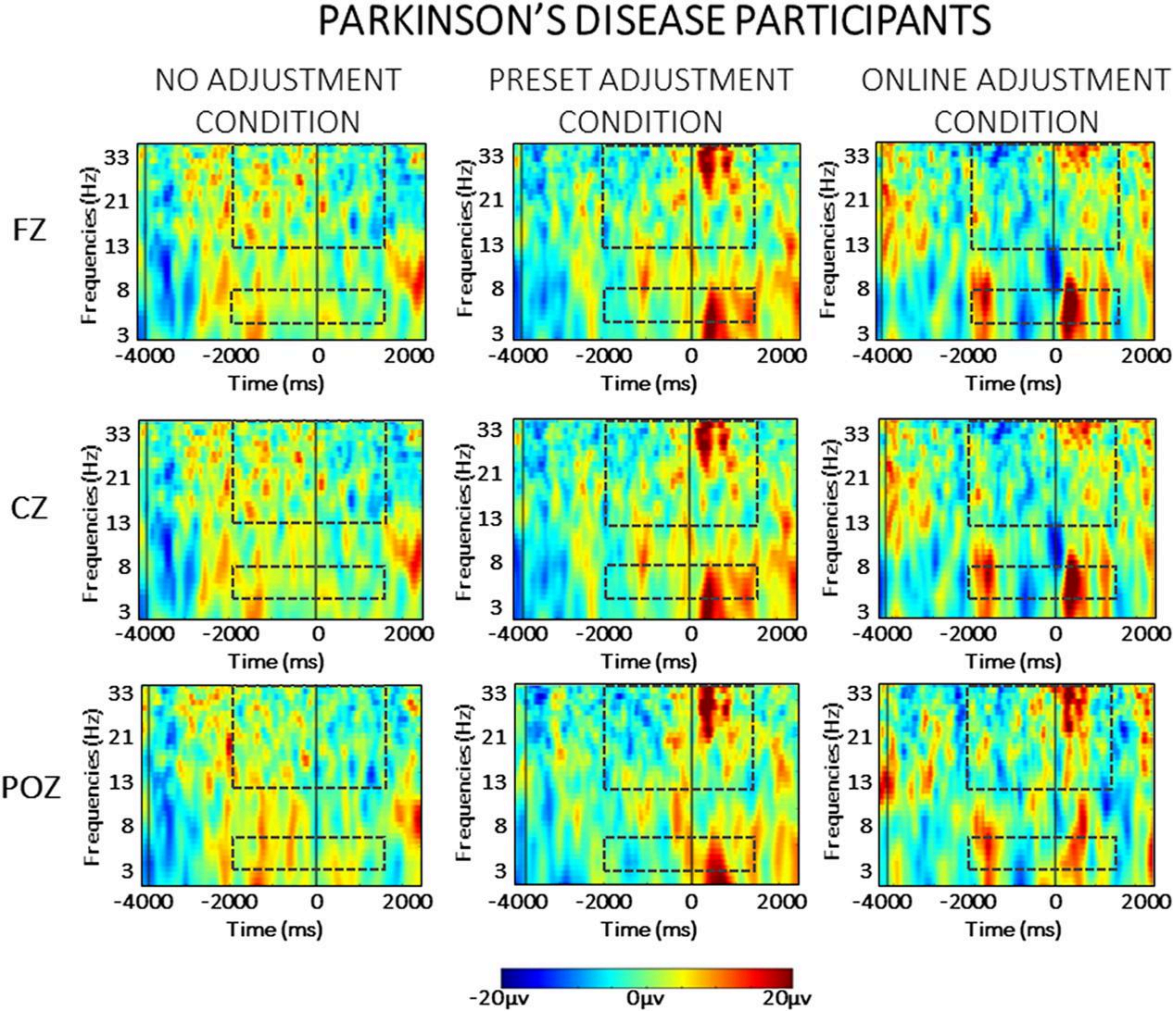
**Averaged time-warped spectrograms of representative midline channels (Fz, Cz and POz) in the Parkinson’s disease group across conditions.** The vertical black lines on the left (around −3000 ms) represent the start of the trial; the vertical black line (at 0 ms) represents the obstacle crossing event. The two black dashed squares in each figure highlight the time and the frequency bands of interest (i.e. theta at 4–7 Hz and beta at 13–35 Hz) in the analysis.

**Figure 5:**
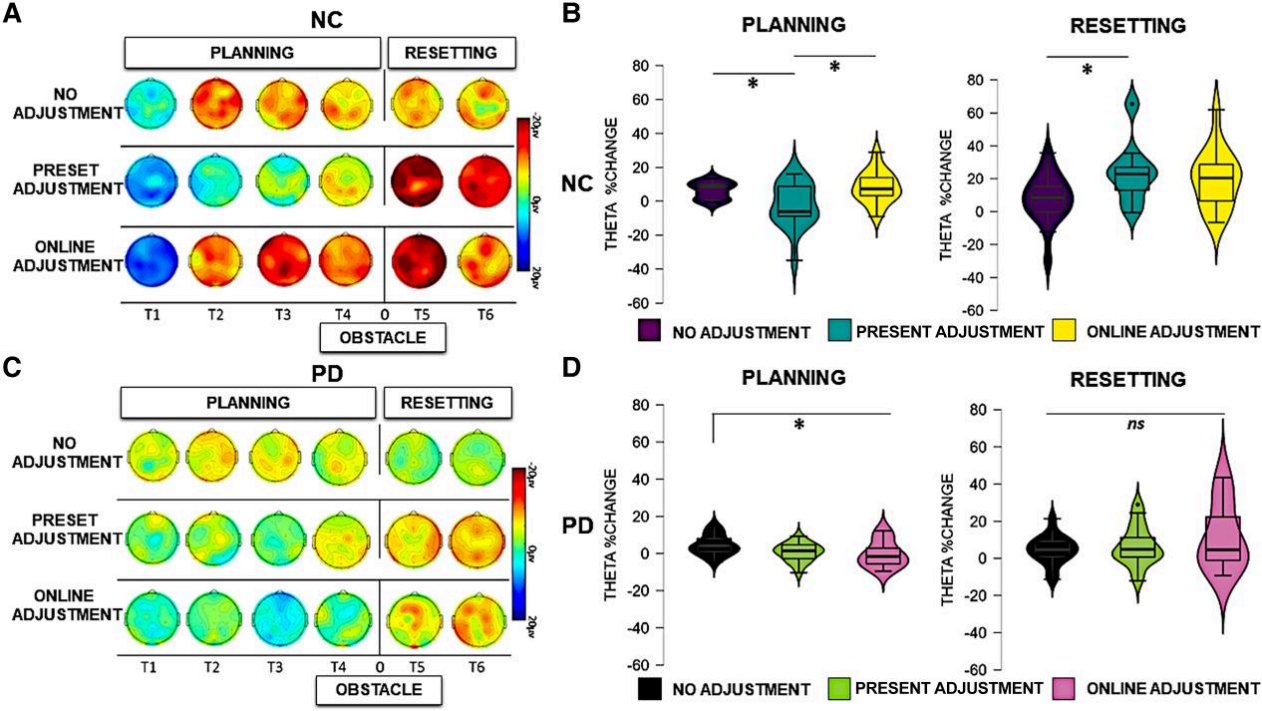
**Theta activity.** Left panels: scalp map topographies in the theta frequency band (4–7 Hz) across conditions and time windows (T1, T2, T3, T4, T5 and T6) in neurotypical control (NC) participants (**A**) and Parkinson’s disease (PD) participants (**C**). Right panels: theta power changes across ROI conditions and time windows in NC participants (**B**) and Parkinson’s disease participants (**D**) during planning and resetting. Significant comparisons, as indicated by the ANOVA and *post hoc* paired sample *t*-tests, are flagged with asterisks. ‘Neurotypical control group’: during planning, there was a stronger relative increase of theta power in the no adjustment (*t* = 2.608, *P* = 0.030) and online adjustment conditions (*t* = 2.595, *P* = 0.020) compared to the preset adjustment condition. During resetting, a stronger relative increase of theta power was present in the preset adjustment condition compared to no adjustment (*t* = 2.902, *P* = 0.030). ‘Parkinson’s disease group’: during planning, there was a stronger relative increase of theta power in the preset adjustment (*t* = 2.757, *P* = 0.016) and online adjustment (*t* = 2.764, *P* = 0.018) conditions compared to no adjustment. During resetting, there were no statistically significant differences in the modulation of theta power across conditions (*P* > 0.05).

**Figure 6:**
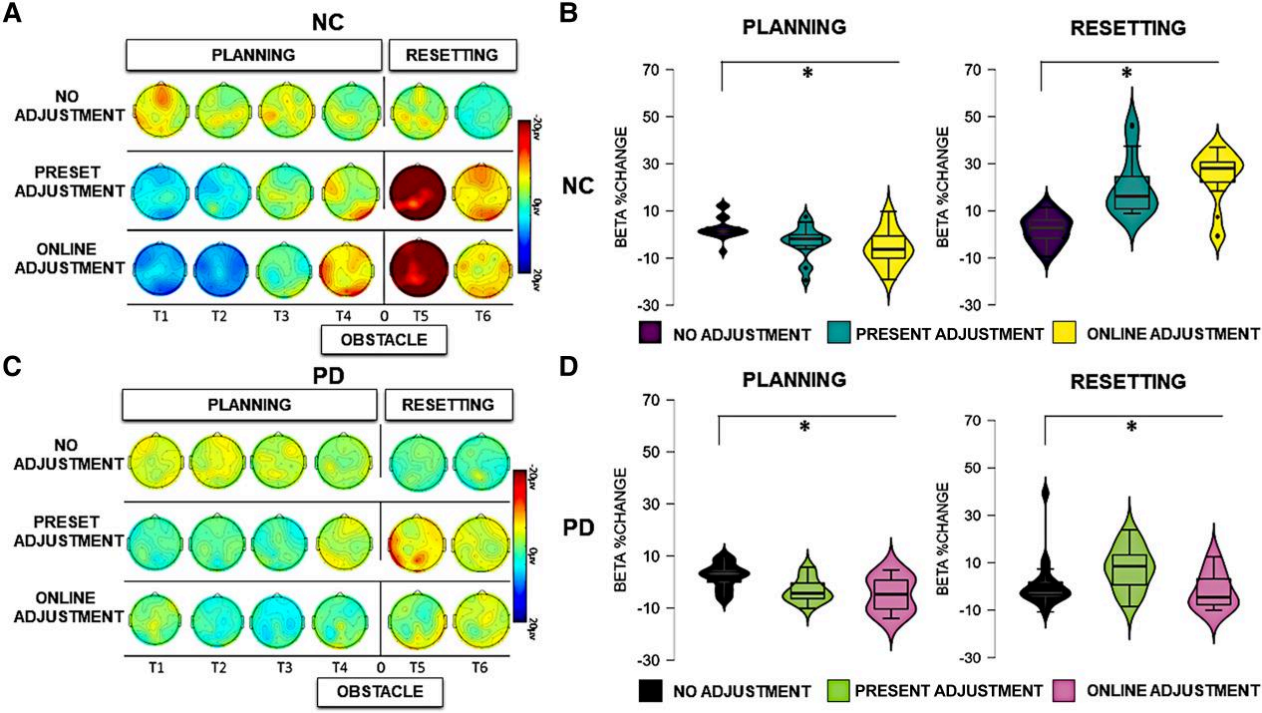
**Beta activity.** Left panels: scalp map topographies in the beta frequency band (13–35 Hz) across conditions and time windows (T1, T2, T3, T4, T5 and T6) in neurotypical control (NC) participants (**A**) and Parkinson’s disease (PD) participants (**C**). Right panels: bar graphs of averaged beta power spectral changes relative to baseline, across ROIs, across conditions (13–35 Hz) in NC participants (**B**) and Parkinson’s disease participants (**D**) during the overall planning and the resetting phase (all time windows). Significant comparisons, as indicated by the ANOVA and *post hoc* paired sample *t*-tests, are flagged with the asterisks. ‘Neurotypical control group’: during planning, there was a stronger relative decrease of beta power in the preset adjustment (*t* = 4.012, *P* < 0.001) and online adjustment (*t* = 4.094, *P*< 0.001) conditions compared to the no adjustment condition. During resetting, a stronger relative increase of beta power was present in the preset adjustment (*t* = 4.311, *P* < 0.001) and online adjustment (*t* = 6.702, *P* < 0.000) conditions compared to the no adjustment condition. ‘Parkinson’s disease group’: during planning, there was a stronger relative decrease of beta power in the preset adjustment (*t* = 3.323, *P* = 0.006) and online adjustment (*t* = 3.711, *P* = 0.003) conditions compared to no adjustment. During resetting, there was a stronger relative increase of beta power in the preset adjustment (*t* = 3.284, *P* = 0.006) and online adjustment (*t* = 3.058, *P* = 0.009) conditions compared to the no adjustment condition.

For the sake of clarity, the results of the statistical analysis of the EEG data are presented separately for the between- and the within-group comparisons across each frequency band (theta and beta) in the two phases of the task (planning versus resetting). As we are interested in evaluating significant differences between groups, only statistically significant interactions with the factor ‘group’ are presented here. For the sake of brevity, all statistical details regarding within-group comparisons are contained in the Supplementary material, as referenced below. Visual depiction of the spectral power in each condition is presented separately for the neurotypical participants (Fig. 3) and for the Parkinson’s participants (Fig. 4), while scalp maps and spectral power changes for both groups are presented for the theta (Fig. 5) and beta (Fig. 6) frequency bands.

Event-related spectral perturbations were computed in the *a priori*–defined frequency ranges, namely theta (4–7 Hz) and beta (13–35 Hz) frequency bands. Below, the results for each frequency band are presented separately for the planning and the resetting phases.

### Planning

#### Theta oscillations

The statistical analysis (mixed ANOVA) of theta power during the planning phase revealed a two-way interaction between group and condition [*F*(1,30) = 4.682, *P* = 0.023, *η*_p_^2^ = 0.168]. *Post hoc* independent sample *t*-tests showed that a stronger relative increase of theta power occurred in neurotypical controls compared to Parkinson’s disease in the online adjustment [*t*(30) = 3.074, *P* = 0.005], as can also be seen in Figs 3 and 4, but not in the no adjustment (*P* = 0.702) or preset adjustment (*P* = 0.426) conditions.

There was a significant two-way interaction between time window and group [*F*(1,30) = 9.370, *P* < 0.001, *η*_p_^2^ = 0.244]. *Post hoc* independent sample *t*-tests showed a stronger relative increase of theta power in neurotypical controls compared to Parkinson’s disease in the first [*t*(30) = 3.224, *P* = 0.003] and in the last time window of the planning phase [*t*(30) = 2.814, *P* = 0.009]. There were no statistically significant differences between the two groups in the second or the third time window of the planning phase (*P* > 0.05).

A significant three-way interaction between time window, condition and group [*F*(1,30) = 2.716, *P* = 0.025, *η*_p_^2^ = 0.087] was further investigated for each time window separately. As illustrated in Fig. 5A and B, during the second time window (corresponding to the time of the appearance of the obstacle in the online adjustment condition), a stronger increase of theta power occurred in neurotypical controls compared to Parkinson’s disease in the online adjustment condition [*t*(30) = 2.829, *P* = 0.008]. Additionally, a stronger relative increase of theta power occurred in neurotypical controls compared to Parkinson’s disease in the online adjustment condition also in the third [*t*(30) = 4.604, *P* < 0.001] and fourth [*t*(30) = 2.942, *P* = 0.006] time windows. *Post hoc* independent sample *t*-tests did not reveal any other statistically significant differences (*P* > 0.05).

A full account of the within-group effects for the Parkinson’s disease group and the control group with regard to theta activity in the planning phase can be found in Supplementary Section S2.1.1.

#### Beta oscillations

The statistical analysis (mixed ANOVA) of beta power during the planning phase revealed a two-way interaction between group and time window [*F*(1,30) = 6.292, *P* < 0.001, *η*_p_^2^ = 0.178]. *Post hoc* independent sample *t*-tests showed a stronger beta power decrease in neurotypical participants compared to participants with Parkinson’s disease in Time Window 1 [*t*(30) = 3.174, *P* = 0.004] and Time Window 4 [*t*(30) = 2.682, *P* = 0.012]. A significant three-way interaction between time window, condition and group [*F*(1,30) = 3.911, *P* = 0.035, *η*_p_^2^ = 0.086] was further investigated in each time window separately. As can be seen in Fig. 6A and B, a stronger relative beta power decrease occurred in the neurotypical controls compared to the Parkinson’s disease group in Time Windows 1 and 2*. Post hoc* independent paired sample *t*-tests revealed a stronger relative decrease of beta power in neurotypical participants compared to participants with Parkinson’s disease during Time Window 1 in both the preset adjustment [*t*(30) = 2.305, *P* = 0.023] and online adjustment [*t*(30) = 3.458, *P* = 0.002] conditions. In Time Window 2, a stronger relative decrease of beta power occurred in the neurotypical controls compared to the Parkinson’s disease group in the online adjustment condition [*t*(30) = 3.275, *P* = 0.003]. *Post hoc* independent sample *t*-tests did not indicate any other statistically significant differences (*P* > 0.05). The ANOVA did not reveal any other main effects or interactions (*P* > 0.05).

A full account of the within-group effects for the Parkinson’s disease group and the control group with regard to beta activity in the planning phase can be found in Supplementary Section S2.1.2. Similar to our previous study,^18^ the within-group analysis revealed a stronger decrease of beta power when neurotypical participants adapted motor plans to step over obstacles, regardless of the expectation manipulation. The within-group analysis revealed that this pattern, likely reflecting motor planning, also occurred in participants with Parkinson’s disease, who exhibited a stronger beta power suppression when stepping over obstacles regardless of the expectation compared to when the participants freely walked the path.

### Resetting

#### Theta oscillations

The between-group effects of the analysis (mixed ANOVA) of theta activity during the resetting phase revealed a main effect of group [*F*(1,30) = 7.518, *P* = 0.010, *η*_p_^2^ = 0.206], reflecting an overall stronger relative increase of theta power in neurotypical participants compared to participants with Parkinson’s disease during the resetting phase, as can be seen in Figs 3 and 4. A four-way interaction [*F*(1,30) = 3.996, *P* = 0.011, *η*_p_^2^ = 0.121] was further investigated in each time window across group and condition separately. As can also be seen in Fig. 5, in Time Window 5, a stronger relative increase of theta power occurred in the neurotypical control group compared to the Parkinson’s disease group in both the preset [*t*(30) = 3.185, *P* = 0.003] and online adjustment [*t*(30) = 2.053, *P* = 0.049] conditions, but not in the no adjustment condition [*t*(30) = 1.602, *P* = 0.120]. *Post hoc* independent sample *t*-tests did not show any statistically significant differences in Time Window 6 (*P* > 0.05).

A full account of the within-group effects for the Parkinson’s disease group and the control group with regard to theta activity in the resetting phase can be found in Supplementary Section S2.2.1. Neurotypical participants exhibited a stronger increase of theta power in the preset adjustment condition compared to no adjustment condition, a difference that was not present when comparing the online adjustment with both the preset and the no adjustment conditions. The analysis also revealed that just after crossing the obstacle, parietal and frontal theta power increases were more pronounced in the preset and in the online adjustment conditions, compared to the no adjustment condition.

#### Beta oscillations

The between-group effects of the analysis (mixed ANOVA) of beta activity during the resetting phase revealed a significant main effect of group [*F*(1,30) = 22.654, *P* < 0.001, *η*_p_^2^ = 0.439], reflecting an overall stronger relative increase of beta power in neurotypical participants compared to participants with Parkinson’s disease. In addition, there was a significant two-way interaction between group and condition [*F*(1,30) = 3.706, *P* = 0.041, *η*_p_^2^ = 0.113]. These effects can also be seen in Figs 3 and 4. *Post hoc* comparisons between groups revealed a stronger relative increase of beta power occurred in neurotypical participants compared to participants with Parkinson’s disease in the no adjustment [*t*(30) = 2.637, *P* = 0.013], preset adjustment [*t*(30) = 2.550, *P* = 0.016] and online adjustment [*t*(30) = 5.164, *P* < 0.000] conditions. To further investigate the two-way interaction, we compared the magnitude of the difference in beta power between groups, across conditions. Paired sample *t*-tests showed that the difference between groups was larger in both the preset and online adjustment conditions compared to the no adjustment condition [no adjustment versus preset adjustment: *t*(30) = 2.212, *P* = 0.042; no adjustment versus online adjustment: *t*(30) = 4.163, *P* < 0.001], whereas there was no difference between the two ‘adjustment’ conditions (*P* = 0.481).

There was also a significant two-way interaction between time window and group [*F*(1,30) = 7.413, *P* = 0.011, *η*_p_^2^ = 0.204]. *Post hoc* comparisons revealed a stronger relative increase of beta power in the neurotypical controls compared to the Parkinson’s disease group in both Time Window 5 [*t*(30) = 4.129, *P* < 0.001] and Time Window 6 [*t*(13) = 3.219, *P* = 0.003], as can be seen in Fig. 6. To further investigate the interaction, we compared the difference in beta power between groups across the two time windows. A paired sample *t*-test showed that the difference in beta power between the two groups was larger in Time 5 compared to the difference in Time Window 6 [*t*(30) = 4.137, *P* < 0.001].

Furthermore, there was a significant two-way interaction between ROI and group [*F*(1,30) = 3.605, *P* = 0.033, *η*_p_^2^ = 0.111]. *Post hoc* comparisons revealed a stronger relative increase of beta power in neurotypical controls compared to Parkinson’s disease group over frontal [*t*(30) = 3.933, *P* < 0.001], central [*t*(30) = 4.556, *P* < 0.001] and parietal [*t*(30) = 5.073, *P* < 0.001] ROIs. Similarly to the previous two-way interactions, differences between groups were compared across ROIs. Paired sample *t*-tests indicated that the difference between groups was larger in the parietal ROI compared both to frontal [*t*(30) = 3.425, *P* = 0.003] and central [*t*(30) = 2.800, *P* = 0.013] ROIs, whereas there was no difference between frontal and central ROIs (*P* = 0.556).

A full account of the within-group effects for the Parkinson’s disease group and the control group with regard to beta activity in the planning phase can be found in Supplementary Section S2.2.2. As we found previously,^17^ a clear beta rebound was found in all conditions, and most strongly after crossing an obstacle, in both the Parkinson’s disease and neurotypical control groups.

## Discussion

The findings of the present study represent an important step towards the understanding of motor–cognitive impairments in Parkinson’s disease. Unlike in previous literature, here we assessed neural markers of cognitive control while participants performed complex natural movements, specifically walking and stepping over obstacles, representing one of the critical challenges that Parkinson’s disease patients face in daily life. Importantly, the temporal resolution of the EEG data allowed the different phases of cognitive processing to be disentangled, highlighting impairments in both proactive and reactive processes, especially when participants with Parkinson’s disease had to adapt to unexpected objects in their path. As the first mobile EEG study to examine the neural correlates of such a dynamic scenario in Parkinson’s disease patients, these results demonstrate that control processes affected by Parkinson’s disease can be investigated during real-world activities. In the following sections, the results of the EEG analysis will be discussed separately for the planning and resetting phase in relation to theta and beta frequency bands. First, however, we summarize the overall pattern of results, highlighting the importance of our findings for understanding the pattern of cognitive and neural impairments observed in Parkinson’s disease, revealing why these patients have difficulties stepping over obstacles.

As expected, behavioural data confirmed that participants with Parkinson’s disease were slower than neurotypical participants when they had to adjust their gait to avoid expected and unexpected obstacles, as well as when they simply walked along the path without obstacles, reflecting the atypical motor patterns commonly observed in Parkinson’s disease.^53^ Furthermore, patients were slower when avoiding obstacles, especially when stepping over unexpected obstacles. More importantly, the neural data revealed that cortical modulations in the theta and beta frequency ranges were attenuated in participants with Parkinson’s disease, both when preparing to step over obstacles (planning phase) and after crossing the obstacles on the floor (resetting phase). Critically, the results suggest that proactive strategies employed to adapt gait when stepping over unexpected obstacles on the floor (reflected in theta power increases) were impaired in Parkinson’s disease. Impairments in motor processes related to the planning of adjustments were also seen in Parkinson’s disease, as indexed by changes in beta power suppression, particularly when online adjustments were required. Furthermore, our data also provided evidence of impaired reactive control mechanisms during the resetting phase, reflected in reduced amplitude in both theta and beta frequency bands in Parkinson’s disease compared to controls. Taken together, these results provide novel insight into the difficulties Parkinson’s disease patients experience when adapting their movement both before and after avoiding an obstacle in their path.

### Planning phase: proactive control mechanisms

As outlined in the introduction, our primary focus concerning the planning phase was to test whether deficits in proactive control were evident in participants with Parkinson’s disease, visible as a theta power increase of smaller amplitude compared to controls during dynamic obstacle avoidance. In the neurotypical group, we replicated the recording of neural markers of proactive control that we reported previously.^17^ The results of the between-group analysis revealed an attenuated theta power increase in participants with Parkinson’s disease compared to neurotypical participants occurring in the condition in which they were required to make online adjustments, but not when walking freely or when they had to adapt the gait to step over an obstacle displayed in advance.

Following our earlier work,^17^ here we also identified a further marker of proactive control (i.e. beta suppression) prior to crossing the obstacle on the floor. The between-group analysis of the present study revealed that stronger power suppression in the beta frequency band occurred in neurotypical participants compared to participants with Parkinson’s disease, in both the preset and online adjustment conditions. We also found condition-specific group differences in beta power limited to early time windows in relation to both obstacle conditions, but as we did not have predictions regarding such complex three-way interactions, these are difficult to interpret. Overall, however, these differences in beta power modulation suggest that participants with Parkinson’s disease present a particular deficit when preparing and selecting motor responses to avoid unexpected obstacles on the floor.

Beta suppression over sensorimotor areas is thought to reflect movement preparation and execution.^54-56^ Indeed, the magnitude of suppression of beta oscillations over the sensorimotor cortex has been related to the selection of appropriate responses.^57-59^ On this basis, the attenuated beta activity observed here when participants with Parkinson’s disease prepared to step over obstacles suggests a deficit in the preparation of appropriate movements in order to adjust their gait and avoid the obstacle. An indication of a similar deficit can be found in studies of non-dynamic finger movements using magnetoencephalography, which found attenuated beta modulation before and during finger movements in participants with Parkinson’s disease compared to controls.^60-62^ For example, Heinrichs-Graham *et al*.^60^ found a reduced beta amplitude during the preparation and the execution of finger tapping movements. Similarly, Stegemöller *et al*.^43,61^ reported attenuated activity during repetitive finger tapping movements that corresponded to the occurrence of hypokinesia and hastening in participants with Parkinson’s disease, suggesting a direct link between beta oscillations and the control of movements.^43^ This neural marker of proactive control, found to be attenuated in Parkinson’s disease in the current study, appears to reflect processes supporting the preparation of gait-adjusted movements that are required to avoid obstacles.

### Resetting phase: reactive control mechanisms

As well as examining planning, our paradigm was also designed to test whether participants with Parkinson’s disease showed compromised motor preparation and reactive control processes during motor adjustments, reflected in attenuated cortical modulation (particularly the beta rebound^55^), compared to neurotypical participants. Critically, during the resetting phase, between-group analysis did reveal that participants with Parkinson’s disease presented an attenuated beta power modulation compared to neurotypical participants in all of our experimental conditions. Notably, however, the diminished beta rebound in Parkinson’s disease was most marked in the two conditions where the patients had to step over an obstacle. The data also revealed differences in distribution during the resetting phase, with the difference between groups being larger over parietal compared to frontal and central electrodes. We have previously established the beta rebound as a marker of reactive control in ambulatory naturalistic obstacle avoidance.^17^ In addition, a diminished beta rebound in Parkinson’s disease patients was previously found in a non-dynamic context, during the proprioception of induced finger movements.^44^ Although the neural source of the beta rebound is commonly reported over central brain areas,^63-65^ several studies have described the involvement of brain areas involved in movement coordination and inhibition, such as the supplementary motor areas, the premotor cortex, the paracentral gyrus and the parietal cortex.^66,67^ Consequently, the beta rebound may reflect both mechanisms related to movement termination and the inhibition of the motor response, which would be mediated by the activation of the parietal cortex.^60,68^ Thus, the differences in the beta rebound between Parkinson’s disease patients and neurotypical controls found in the present study suggest that processes related to the resetting of motor programmes and motor inhibition are affected in Parkinson’s patients.

Finally, while we did not have specific *a priori* hypotheses about differences in theta during the resetting phase, the data revealed that straight after crossing an obstacle, Parkinson’s disease patients differed from controls. More specifically, the analysis revealed a smaller theta power increase in the Parkinson’s disease group compared to neurotypical participants. Theta oscillations have previously been found to signal both proactive and reactive control mechanisms.^69,70^ For example, Eschmann *et al*.^69^ reported theta power increases during tasks requiring either proactive (delayed match to sample task) or reactive (Stroop task) control strategies. Theta power increases have also been observed during the evaluation of action outcomes in a forced choice speeded response and reinforcement learning tasks.^70,71^ This evidence, taken together with the pattern of group differences in theta during the resetting phase found here, indicates that theta oscillations might signal the evaluation of the action outcomes during real-world obstacle avoidance and that this action outcome evaluation is affected by Parkinson’s disease.

### Treatment considerations

It should be noted that one potentially important feature of the current study was that participants with Parkinson’s disease were tested while on medication, i.e. 1–2 h after the administration of dopamine. Several EEG studies have suggested a specific link between dopamine and cortical beta power.^72-74^ For example, Melgari *et al*.^72^ demonstrated that the administration of dopaminergic treatment increased the level of beta modulation over central and parietal sites in Parkinson’s disease patients during resting state EEG recordings. Similarly, Mostile *et al*.^73^ found the dopaminergic administration increased beta over frontal areas in untreated Parkinson’s disease patients, and George *et al*.^74^ found increased beta over frontal and sensorimotor areas in Parkinson’s disease patients ON compared to OFF medication while performing a stop–signal task. There is, by contrast, limited evidence in relation to dopaminergic effects on the beta rebound effect. For example, Vinding *et al*.^62^ did not find any difference between groups of Parkinson’s disease patients in ON versus OFF medicated states and showed a similar attenuated beta rebound related to finger movements in both groups of patients compared to NC.

While these previous findings suggest that dopaminergic medication can affect beta modulations, and medication therefore could have influenced the pattern of beta modulations seen here in participants with Parkinson’s disease, controlled medication was essential due to the demanding nature of the task for the patients, which prevented assessment in OFF state. Although testing during ON compared to OFF medication would of course be of value, the feasibility of this when evaluating dynamic real-world ambulatory movements in Parkinson’s disease is questionable, even in the moderate stage of Parkinson’s disease. Nonetheless, future research is clearly needed to further investigate whether dopaminergic medications can restore cortical activity in Parkinson’s disease during the preparation of motor adaptations, especially in daily living activities, such as during real-world obstacle avoidance.

As noted above, the findings of the present study represent an important step towards the understanding of motor–cognitive impairments in Parkinson’s disease with regard to the kind of complex natural movements that pose the most critical challenges that Parkinson’s disease patients face in daily life. Crucially, the results showed that cognitive impairment was not directly related to dopaminergic depletion, since the participants with Parkinson’s disease recruited for the present study were on a stable medication regime and medication was controlled with respect to the time of testing. This is in line with previous behavioural evidence, which suggests that dopamine might improve motor functions but differently impact components of cognitive control.^75-77^ For example, Duthoo *et al*.^75^ found that Parkinson’s disease patients in ON state did not exhibit adaptation to conflict in the Stroop task compared to patients in OFF medication state, suggesting that dopamine might be detrimental to cognitive conflict. Conversely, Ruitenberg *et al*.^76^ found that medication status did not affect conflict adaptation during the Stroop task in Parkinson’s disease patients but improved motor performance.

Taken together, previous literature and the present results provide important new insight into the involvement of cortically mediated cognitive control processes in the pathophysiological mechanism of the disorder and suggest new targets for the pharmacological treatments and refined ways of evaluating their efficacy. The neural specificity of the identified cognitive markers, and their temporal resolution, opens up potential new rehabilitation strategies for Parkinson’s disease such as brain stimulation and/or neurofeedback (as previously applied in stroke rehabilitation, see Schlaug *et al*.^78^). It would, however, require further research to establish whether ‘reintroduction’ of targeted brain oscillations through such rehabilitation techniques would also bring the desired behavioural improvements. For example, future studies could investigate the link between brain signals related to obstacle avoidance and the accuracy of the behavioural performance, which could not be explored with the present setup.

Detailed behavioural measures may, for example, be particularly important for characterizing different Parkinson’s disease subtypes. Indeed, the association between cortical activity and behavioural performance in Parkinson’s disease might be specific to motor impairments and task dependent.^79^ Several studies^80-82^ have reported reduced activity over prefrontal brain areas in Parkinson’s disease patients during turning but increased activity in patients with freezing of gait.^80,81^ These findings suggest that in the presence of major gait disturbances, more cognitive and cortical resources are needed to compensate for motor impairment.^81^ Conversely, there is also evidence of reduced cortical activity in Parkinson’s disease during simple gait tasks, such as step initiation compared to standing.^82^ The different patterns of results reported within the wider literature most likely reflect variability in both task requirements and the specific motor impairment being investigated. The inclusion of behavioural data in future studies is therefore critical for understanding whether changes in cortical activity reflect the motor system’s attempt to compensate and restore subcortical motor processes compromised in Parkinson’s disease.^79^

## Conclusion

Compared to neurotypical participants, the data reveal that participants with Parkinson’s disease showed attenuated theta and beta power modulation when planning and implementing a motor adaptation to step over obstacles displayed on the floor, particularly when the obstacles were unexpected. These findings provide neural evidence that Parkinson’s disease compromises patients’ abilities to monitor and flexibly modify their actions when the environment presents unexpected challenges in the context of real-world ambulation. In particular, the results showed that when encountering an obstacle on the floor, participants with Parkinson’s disease exhibited deficits in both proactive (visible in attenuated theta power increase) and reactive (visible in a reduced beta rebound) control mechanisms. Taken together, the present data are suggestive of a pervasive deficit in motor–cognitive control processes in Parkinson’s disease, which, in real-world scenarios such as when walking and avoiding objects on the floor, might have a significant impact on the risk of falling and hospitalization. Finally, further to our previous work in young healthy volunteers,^17^ the present study demonstrates the clinical utility of mobile EEG capturing neural markers of motor–cognitive control in real-world contexts. Therefore, the innovative approach taken in this study is a first step in providing a neuroscientific basis for novel rehabilitative techniques that are more meaningful for Parkinson’s patients’ daily life and furthermore provides a basis for the development of more ecologically valid tools for drug evaluation.

## Supplementary Material

fcad326_Supplementary_Data

## Data Availability

The anonymized data of the present study are available at https://osf.io/fxb8z/.
