# Supplementary material for: Characterizing neurocognitive impairments in Parkinson’s disease with mobile EEG when walking and stepping over obstacles
Source: Brain Commun. 2023 Nov 28;5(6):fcad326. doi: 10.1093/braincomms/fcad326 (PMC10724048; doi:10.1093/braincomms/fcad326)
Supplement: fcad326_Supplementary_Data [file fcad326_supplementary_data.docx]

**Supplementary Materials**

**S1. Methods**

**S1.1 Additional information**

PD group inclusion criteria were: age ≥ 50; diagnosis of idiopathic PD; absence of cognitive impairments (MMSE score ≥ 24); absence of depression; absence of neurological or relevant comorbidities; able to perform the Time Up and Go test; able to walk without aid or assistance for more than 5 minutes. NC group inclusion criteria were: age ≥ 50; absence of cognitive impairments (MMSE score ≥ 24); absence of any neurological condition.

The resulting sample size of this study was largely determined by the ability to walk without aid or assistance for more than 5 minutes, which meant that many of the population that we recruited from were not able to cope with the requirements of the set up. The coronavirus pandemic at the time allowed for no flexibility with regards to the recruitment period, nor with regards to the recruitment of controls. Nonetheless, despite these constraints, it is notable that the resulting sample size is in keeping with the sample size of other relevant studies^1,2,3^.

Stimulus presentation was controlled using E-prime 3.0 software (Psychology Software Tools) and a projector. The motion sensors were designed to send an input signal to the stimulus presentation software running on a laptop when the sensors were crossed by the participants using the Auxiliary I/O port of a Chronos response device (Psychology Software Tools). The laptop was connected to a projector placed at the side of the carpet. The presence and location of the obstacle varied on a trial-by-trial basis, depending on the experimental condition. During each trial, the onset of walking (‘Start’), the passage through the motion sensors (‘Approach’), the obstacle avoidance (‘Crossing’) and the cessation of walking (‘Stop’) were recorded manually by the experimenter to timestamp the EEG data. These time points provided temporal markers for the EEG data, that identified a planning phase (before the obstacle was encountered, from the ‘Approach’ to ‘Crossing’) and a resetting phase (after the obstacle was encountered, from ‘Crossing’ to the ‘Stop’).

**S1.2 EEG acquisition and pre-processing**

EEG data were recorded from 32 Ag/AgCl electrodes connected to a portable amplifier (ANT-neuro, Enschede, The Netherlands). Electrodes were positioned according to the International 10–20 system (FP1, FPz, FP2, F7, F3, Fz, F4, F8, FC5, FC1, FC2, FC6, M1, T7, C3, Cz, C4, T8, M2, CP5, CP1, CP2, CP6, P7, P3, Pz, P4, P8, POz, O1, Oz, O2) with AFz electrode as ground and CPz electrode as reference. The electrode impedances were reduced below 5 kΩ before the recording. During recording, EEG data were sampled at 500 Hz and bandpass filtered at 0.01–250 Hz. EEG data analyses were performed using custom scripts written in MATLAB 2019a (The MathWorks) incorporating EEGLAB toolbox^4^. First, data from the mastoid channels (M1 and M2) were removed from the analysis, and all remaining EEG data was filtered using a 0.1 Hz to 80 Hz bandpass filter. EEG channels with prominent artifacts were automatically identified (kurtosis > 5 SDs) and interpolated, and all channels were then re-referenced to the average. Data were down sampled to 250 Hz and an extended infomax Independent Component Analysis (ICA^5^) was performed to identify and remove non-brain signals. Brain-related and non-brain ICs were identified using the IClabel plugin^6^.

**S2. Within group effects of the EEG analysis**

**S2.1 Planning**

**S2.1.1 Theta**

**Within group effects.** NC group: The ANOVA revealed a main effect of Condition [F(1, 16) = 6.248, p = .016, *η_p_^2^* = .281, Figure 4 of the mean text], indicating a clear overall relative increase of theta power in the no adjustment condition and in the online adjustment conditions compared to the preset adjustment condition [no adjustment vs preset adjustment: t(16) = 2.608, p = .019; online adjustment vs preset adjustment: t(16) = 2.595, p = .020] conditions, but no differences between the online adjustment and the no adjustment conditions (p = .278). A main effect of Time Window [F(1, 16) = 19.885, p < .001, *η_p_^2^* = .554] showed a stronger increase of theta power relative to the baseline during Time 1 compared to the other time windows [Time 1 vs Time 2: t(16) = 5.837, p < .001; Time 1 vs Time 3: t(16) = 5.478, p < .001; Time 1 vs Time 4: t(16) = 5.298, p < .001] and in second Time Window compared to the third [Time 2 vs Time 3: t(16) = 3.204, p = .006]. A significant 2-way interaction between Time Window and Condition [F(1, 30) =3.610, p = .016, *η_p_^2^* = .184] showed that a stronger relative increase of theta power occurred in Time 3 in the no adjustment condition compared to the preset adjustment condition [t(16) = 3.169, p = .006], and in the online adjustment condition compared to the preset adjustment condition [t(16) = 4.505, p < .001]. The ANOVA and post-hoc paired sample t-tests did not show any other statistically significant differences (p > .05).

PD group: The ANOVA revealed a main effect of Condition [F(1, 13) = 4.724, p = .022, *η_p_^2^* = .267, Figure 4 of the main text], reflecting a stronger relative increase of theta power in the preset adjustment and online adjustment conditions compared to no adjustment [no adjustment vs preset adjustment: t(13) = 2.757, p = .016; no adjustment vs online adjustment: t(13) = 2.764, p = .018], but no statistically significant differences between preset adjustment and online adjustment conditions (p > .05). The ANOVA did not reveal any other statistically significant main effects or interactions (p > .05).

**S2.1.2 Beta**

**Within group effects.** NC group: The ANOVA revealed a main effect of Condition [F(1, 16) = 9.427, p < .001, *η_p_^2^* = .371, Figure 5 of the main text], indicating a stronger relative decrease of beta power in the preset adjustment and online adjustment conditions compared to the no adjustment condition [no adjustment vs preset adjustment: t(16) = 4.012, p < .001; no adjustment vs online adjustment: t(16) = 4.094, p < .001] but no differences between the preset and the online adjustment conditions (p = .335). A main effect of Time Window [F(1, 16) = 9.492, p < .001, *η_p_^2^* = .372] indicated that a stronger relative decrease of beta power occurred during Time 1, Time 2 and Time 3 compared to Time 4 [Time 1 vs Time 4: t(16) = 4.496, p < .001; Time 2 vs Time 4: t(16) = 3.472, p = .003; Time 3 vs Time 4: t(16) = 3.787, p = .012].

A significant 2-way interaction between Time Window and Condition [F(1, 16) = 4.157, p = .007, *η_p_^2^* = .206] showed a stronger relative decrease of beta power in the preset adjustment and in the online adjustment conditions compared to no adjustment in both Time 1 [no adjustment vs preset adjustment: t(16) = 3.168, p = .006; no adjustment vs online adjustment: t(16) = 5.039, p < .001] and Time 2 [no adjustment vs preset adjustment: t(16) = 3.315, p = .004; no adjustment vs online adjustment: t(16) = 5.675, p < .001]. The ANOVA did not reveal any other main effects or interactions (p > .05).

PD group: The ANOVA revealed a main effect of Condition [F(1, 13) = 9.613, p < .001, *η_p_^2^* = .425, Figure 5 of the main text], indicating a stronger relative decrease of beta power in the preset adjustment and online adjustment conditions compared to the no adjustment condition [no adjustment vs preset adjustment: t(13) = 3.323, p = .006; no adjustment vs online adjustment: t(16) = 3.711, p = .003] but no differences between the preset and the online adjustment conditions (p = .145). The ANOVA did not show any other significant main effects or interactions (p > .05).

**S2.2 Resetting**

**S2.2.1 Theta**

**Within group effects.** NC group: The ANOVA revealed a main effect of Condition [F(1, 16) = 6.040, p = .010, *η_p_^2^* = .274, Figure 4 of the main text]. Post hoc paired sample t-tests confirmed a stronger relative increase of power was present in the preset adjustment condition compared to no adjustment [t(16) = 2.902, p = .030], but did not indicate any other statistically significant differences between conditions (p > .05). A main effect of Time Window [F(1, 16) = 8.330, p = .011, *η_p_^2^* = .342] showed that a stronger relative increase of theta power occurred just after the crossing point (Time 5). A significant 3-way interaction [F(1, 16) = 3.777, p = .025, *η_p_^2^* = .191] was investigated for each Time window separately. In Time window 5 a stronger relative increase of theta power occurred in both the preset and in the online adjustment conditions compared to no adjustment over frontal [no adjustment vs preset adjustment: t(16) = 3.253, p = .005; no adjustment vs online adjustment: t(16) = 4.277, p < .001] and parietal areas [no adjustment vs preset adjustment: t(16) = 3.477, p = .003; no adjustment vs online adjustment: t(16) = 3.232, p = .005]. Post-hoc independent sample t-tests did not indicate any other statistically significant differences in Time 6 (p > .05).

PD group: The ANOVA did not reveal any statistically significant main effects or interactions (p < .05, Figure 4 of the main text).

**S2.2.2 Beta**

**Within group effects.** NC group: The ANOVA revealed a main effect of Condition [F(1, 16) = 16.125, p < .001, *η_p_^2^* = .502, Figure 5 of the main text] reflecting a stronger relative increase of beta power in both the preset and online adjustment conditions compared to the no adjustment condition [no adjustment vs preset adjustment: t(16) = 4.311, p < .001; no adjustment vs online adjustment: t(16) = 6.702, p < .001], but no differences between the preset and the online adjustment conditions (p = .929). A main effect of Time Window [F(1, 16) = 18.953, p < .001, *η_p_^2^* = .542] indicated a stronger relative increase of beta power in the time window after the crossing point (Time 5). A main effect of ROI [F(1, 16) = 7.215, p = .007, *η_p_^2^* = .11] indicated that a stronger increase of beta power occurred over parietal compared to central [t(16) = 3.224, p = .005] and frontal [t(16) = 4.314, p < .001] ROIs. A significant 2-way interaction between Time Window and Condition [F(1, 16) = 4.530, p = .023, *η_p_^2^* = .221] showed that beta power increase relative to the baseline was stronger in the preset and online adjustment conditions compared to the no adjustment condition in both Time 5 [no adjustment vs preset adjustment: t(16) = 3.444, p = .003; no adjustment vs online adjustment: t(16) = 5.178, p < .001] and Time 6 [no adjustment vs preset adjustment: t(16) = 2.988, p = .009; no adjustment vs online adjustment: t(16) = 2.496, p = .009]. A significant 2-way interaction between Time Window and ROIs [F(1, 16) = 4.340, p = .033, *η_p_^2^* = .213] showed that a stronger relative increase of power occurred in Time 5 over parietal compared to central [t(16) = 3.624, p = .002] and frontal [t(16) = 3.826, p < .001] ROIs, but no differences in Time 6.

PD group: The ANOVA revealed a main effect of Condition [F(1, 13) = 4.769, p = .025, *η_p_^2^* = .268], indicating a stronger relative increase of beta power occurred in both the preset adjustment and online adjustment conditions compared to the no adjustment condition [no adjustment vs preset adjustment: t(16) = 3.284, p = .006; no adjustment vs online adjustment: t(13) = 3.058, p = .009], but no differences between the preset and online adjustment conditions (p = .266). The ANOVA did not show any other significant main effects or interactions (p > .05).

**Supplementary references**

1. Wu HM, Hsiao FJ, Chen RS, Shan DE, Hsu WY, Chiang MC, & Lin YY. Attenuated NoGo-related beta desynchronisation and synchronisation in Parkinson’s disease revealed by magnetoencephalographic recording. *Scientific reports*, 2019; 9(1), 1-12

2. Stegemöller EL, Allen DP, Simuni T, MacKinnon CD. Motor cortical oscillations are abnormally suppressed during repetitive movement in patients with Parkinson’s disease. *Clinical Neurophysiology*. 2016;127(1):664-674. doi:10.1016/j.clinph.2015.05.014

3. Heinrichs-Graham E, Wilson TW, Santamaria PM, Heithoff SK, Torres-Russotto D, Hutter-Saunders JA, Estes KA, Mosley RL, & Gendelman HE. Neuromagnetic evidence of abnormal movement-related beta desynchronization in Parkinson's disease. *Cerebral cortex*, 2014; 24(10), 2669-2678

4. Brunner C, Delorme A, Makeig S. Eeglab – an Open Source Matlab Toolbox for Electrophysiological Research. *Biomedical Engineering / Biomedizinische Technik*. Published online January 7, 2013. doi:10.1515/bmt-2013-4182

5. ‌Makeig S, Jung TP, Ghahremani D, & Sejnowski TJ. Independent component analysis of simulated ERP data. *Institute for Neural Computation, University of California: technical report INC-9606*. 2004

6. Pion-Tonachini L, Kreutz-Delgado K, Makeig S. ICLabel: An automated electroencephalographic independent component classifier, dataset, and website. *NeuroImage*. 2019;198:181-197. doi:10.1016/j.neuroimage.2019.05.026
